# A Systematic Review of Bisphenol A from Dietary and Non-Dietary Sources during Pregnancy and Its Possible Connection with Fetal Growth Restriction: Investigating Its Potential Effects and the Window of Fetal Vulnerability

**DOI:** 10.3390/nu13072426

**Published:** 2021-07-15

**Authors:** Nikolaos Vrachnis, Nikolaos Loukas, Dionysios Vrachnis, Nikolaos Antonakopoulos, Dimitrios Zygouris, Aggeliki Kοlialexi, Vasilios Pergaliotis, Christos Iavazzo, George Mastorakos, Zoi Iliodromiti

**Affiliations:** 1 3rd Department of Obstetrics and Gynecology, Medical School, National and Kapodistrian University of Athens, Attikon Hospital, 12462 Athens, Greece; nloux13@hotmail.com (N.L.); antonakopoulos2002@yahoo.gr (N.A.); zyg14@hotmail.com (D.Z.); akolial@med.uoa.gr (A.K.); 2 Vascular Biology, Molecular and Clinical Sciences Research Institute, St George’s University of London, London SW17 0RE, UK; 3 Endocrinology Unit, 2nd Department of Obstetrics and Gynecology, Medical School, National and Kapodistrian University of Athens, Aretaieio Hospital, 11528 Athens, Greece; dionisisvrachnis@gmail.com (D.V.); mastorakg@gmail.com (G.M.); 4 Gynecology, Obstetrics and Perinatal Medicine Unit, Evgenideio Hospital, National and Kapodistrian University of Athens, 11528 Athens, Greece; 5 1st Department of Obstetrics and Gynecology, National and Kapodistrian University of Athens, Alexandra Hospital, 11528 Athens, Greece; pergialiotis@hotmail.com; 6 Gynaecological Oncology Department, Metaxa Cancer Hospital, 18537 Piraeus, Greece; christosiavazzo@hotmail.com; 7 Department of Neonatology, Medical School, National and Kapodistrian University of Athens, Aretaieio Hospital, 11528 Athens, Greece; ziliodromiti@yahoo.gr

**Keywords:** bisphenol A, food, food packaging, endocrine-disrupting chemical, fetal growth restriction, birth weight, small for gestational age

## Abstract

Bisphenol A (BPA), a ubiquitous endocrine-disrupting chemical (EDC), is increasingly hypothesized to be a factor contributing to changes in fetal growth velocity. BPA exposure may be environmental, occupational, and/or dietary, with canned foods and plastic bottles contributing significantly. Our systematic review aims to evaluate the current literature and to investigate the role of BPA in abnormal fetal growth patterns. A search was conducted in the PubMed and Cochrane databases. A total of 25 articles met the eligibility criteria and were included in this systematic review. Eleven of them failed to show a clear relationship between BPA and abnormal fetal growth. The majority of the remaining studies (9/14) found an inverse association of BPA with indicators of fetal growth, whereas three studies suggested increased fetal growth, and two studies produced contradictory findings. Of note, both of the studies that collected a sample (amniotic fluid) directly reflecting BPA concentration in the fetus during the first half of pregnancy revealed an inverse association with birth weight. In conclusion, there is mounting evidence that combined exposure to BPA from dietary and non-dietary sources during pregnancy may contribute to abnormal fetal growth; a tendency towards fetal growth restriction was shown, especially when exposure occurs during the first half.

## 1. Introduction

Bisphenol A (BPA) is a chemical substance possessing endocrine-disrupting properties with the capacity to affect normal hormone homeostasis mainly due to its estrogen-mimicking mechanisms and its interaction with thyroid and androgen hormones [1]. These effects are mediated through its interference with several nuclear and non-nuclear receptors and enzymatic pathways involved in steroidogenesis. In particular, BPA has the ability to bind with estrogen, estrogen-related transcription, androgen, and thyroid hormone receptors, which regulate hormones essential for proper fetal development. It can also act as a ligand for the pregnane X receptor and the aryl hydrocarbon receptor, which are involved in the regulation of xenobiotic metabolism, participating in key detoxification processes. Activation of the aryl hydrocarbon receptor may also contribute to the upregulation of cytochrome P450 1A1 enzyme (CYP1A1) activity, which has been associated with reduced fetal growth velocity [2]. Moreover, BPA interaction with peroxisome proliferator-activated receptors can affect adipogenesis, while altered cytochrome P450 family 11 subfamily A member 1 (CYP11A1), cytochrome P450 family 19 (CYP19), and steroidogenic acute regulatory protein (StAR) expression and ERK activity may provoke a downregulation of steroidogenesis [3,4]. BPA can also impair fetal growth by disrupting the placenta’s crucial role in regulating nutrient transport. A number of studies have demonstrated downregulation in human placenta of ATP-binding cassette subfamily G member 2 (ABCG2) protein, which protects the fetus from toxic substances [5], while in mice, abnormal placentation and trophoblast invasion were identified [6].

Moreover, this endocrine-disrupting chemical (EDC) exerts both genotoxic and epigenotoxic effects. BPA can contribute to epigenetic changes, such as DNA methylation, genomic imprinting, histone modifications, and altered micro-RNA expression [3,7]. Ιn human trophoblast cells, decreased methylation of genes influencing metabolic and oxidative stress has been demonstrated [8]; meanwhile, in mice, it has been observed that wingless-type MMTV integration site family member 2 (WNT2) expression is downregulated through the methylation of DNA [6]. These epigenetic modifications have been associated with a higher risk for fetal growth restriction (FGR), as they are involved in stress pathways and in angiogenic and endocrine function [9,10]. In addition, altered placental micro-RNA expression has the potential to affect fetal growth by affecting placental development and function [11,12]. Finally, as regards its genotoxicity, BPA has been associated with chromosomal aberrations and impaired meiotic progress, while it also disrupts double-strand break repair. Importantly, some of these changes can be retained across generations and be inherited by offspring [3,13].

Exposure to BPA can be divided into dietary (through food consumption and food contact material) and non-dietary (environmental and occupational. e.g., inhalation of air and dust, dermal contact (thermal paper, clothing, toys, personal care products, water pipes, medical equipment), soil, etc.). However, the main sources are through the diet given that it can leach into food and water/drinks via protective food coatings and plastic bottles made from BPA, mainly when these are exposed to heat or are overused. Specifically, BPA is used to manufacture polycarbonate plastics and epoxy resins and is present in inter alia, plastic containers and bottles, food can coatings, and food packaging. BPA has therefore been detected in such canned foods as condiments, vegetables, dairy and meat products, beverages, fish, and seafood [14,15]. 

The ubiquitous exposure of humans to endocrine disruptors (EDs) has been associated with disruption of reproductive health and fertility problems, while prenatal exposure has been linked to, among others, fetal neurodevelopmental health and growth restriction [16,17]. These effects have raised concerns over the past few decades regarding the ability of this substance to nullify the beneficial role of nutrients in foods such as vegetables and milk and to impede the nutrient absorption of vegetables cultivated in soil contaminated with BPA. As a result, many countries have banned the use of BPA in products intended for infants and young children [18]. The present-day perspective of the US Food and Drug Administration is that current levels of BPA in food can be considered safe [19]. While the European Food Safety Authority (EFSA) reached the same conclusion in 2015, in 2018, a new group of experts was assembled to re-evaluate the potential hazardous effects shown in recent toxicological studies, with the final results being awaited [20]. However, since exposure solely through food may largely be deemed safe while overall exposure could well cause adverse effects, BPA has been replaced in a wide array of products by analogs, such as bisphenol S (BPS), bisphenol F (BPF), and bisphenol B (BPB), which are considered to be safer for human health [21]. 

**Figure 1:**
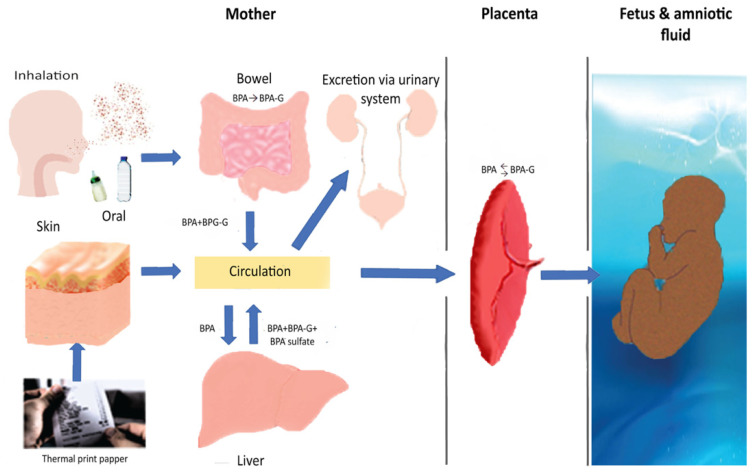
Bisphenol A metabolism. In adults, bisphenol A is mainly metabolized to BPA-glucuronide (BPA-G) in the liver, and a smaller proportion in the intestines, via UDP-glucuronyltransferase. Hepatic sulfation is another pathway (BPA sulfate via sulfotransferase). The metabolites are excreted in urine. They are inactive, but they can be deconjugated and return to the active form via β-glucuronidase (GUSB) and steroid sulfatase (STS). Skin contact seems to lead to longer exposure to free BPA. During pregnancy, BPA can pass through the placenta. The fetus is theoretically more vulnerable because of absent or reduced UDP-glucuronyltransferase, especially during the first two trimesters. It has also been proposed that it is capable of deconjugation, and the placenta can contribute to this procedure, thereby increasing the active form. Bisphenol A concentrations are the same (a) in the blood vessels in the placenta that carry fetal blood, (b) in the umbilical cord, and (c) in the blood circulation in the fetus.

Nevertheless, crucially, scientific concerns regarding BPA toxicity in fetuses, infants, and children have in recent years been on the rise. BPA has been found in many fetal biological samples, including cord blood and amniotic fluid, despite its short half-life and high variability, thus showing that it can cross the placental barrier [22,23]. Moreover, exposure during pregnancy or early childhood has been associated with a large number of fetal and perinatal adverse effects, including decreased growth velocity, preterm birth, recurrent miscarriages, and reduction of the anogenital distance among boys, with data on its suspected link to preeclampsia still being scarce [24]. Prenatal and childhood exposure has been also linked to neurobehavioral problems, expressed as increased levels of anxiety, depression, and hyperactivity [25]. Finally, exposure during gestation has been associated with childhood asthma, wheezing, obesity, metabolic disorders, and damage to the reproductive system [26,27]. BPA metabolism is illustrated in Figure 1 [28,29]. 

While numerous studies have been conducted on the potential association of BPA with fetal growth, it is evident that a still fuller understanding of the exact way in which it may impair intrauterine development is of utmost importance due to the significance of the prenatal period for infancy, childhood, adolescence, and adult life [30]. Fetal growth restriction (FGR) refers to fetuses that have not achieved their true growth potential and present with reduced growth velocity. Small for gestational age (SGA) refers to fetuses/neonates that have an estimated fetal weight (EFW) or birth weight (BW) less than the 10th centile for gestational age. However, all FGR fetuses are not SGA, while SGA fetuses/neonates do not necessarily reflect pathology, as many of them are constitutionally small based on the mother’s characteristics and ethnicity. There are also other indices used for the evaluation of proper growth, such as the ponderal index, which determines the relationship between the mass and height of the neonate and low birth weight (LBW, i.e., an infant weighing less than 2500 g). Intrauterine growth is evaluated through the ultrasound (US) biometry results (a combination of measurement of abdominal and head circumference and femur length) and Doppler measurements of uterine, umbilical and middle cerebral artery, and the ductus venosus [31]. Growth-restricted fetuses/neonates show increased perinatal morbidity and mortality and are at increased risk for a number of adverse outcomes in both the prenatal period and at all subsequent stages of life, as aforementioned [32,33]. Investigation into factors that may contribute to these conditions and, most importantly, research into how exposure to them can be avoided will help in the prevention of such long-term adverse outcomes.

Our aim is to present a systematic review of the current literature and to examine the ability of BPA to impair normal fetal growth while simultaneously investigating whether a specific period of increased susceptibility exists.

## 2. Materials and Methods

In order to write this review, we followed the PRISMA statement recommendations. We did not register a protocol for this systematic review, as there was no previous publication registered or issued during our research procedure.

### 2.1. Search Strategy

To conduct our review, we searched the PubMed (Medline, Baltimore, MD, USA) and Cochrane databases for articles, from their conception to September 2019. The search terms used were “bisphenol A birth weight” and “bisphenol A fetal growth”.

### 2.2. Eligibility Criteria

The criteria were decided upon by the researchers. We included all identified epidemiological studies that investigated the association of BPA with human fetal growth, provided that they measured BPA in a biological sample at least once during pregnancy or delivery. Further, only studies that used birth weight, ponderal index, SGA, LBW, or US parameters (head or abdominal circumference, femoral length, or estimated fetal weight) as indicators of fetal growth were included. Birth weight was the main outcome used as the indicator of intrauterine growth in most of the studies. We excluded all reviews, animal studies, and articles not written in the English language. Furthermore, we excluded all studies that did not measure BPA during pregnancy or at delivery or did not measure it in a biological sample.

### 2.3. Study Selection Progress

Two researchers screened the published articles based on the eligibility criteria mentioned above. The full text of the studies that were not excluded by title or abstract was examined. The researchers were not blinded to the study title or authors and the data were extracted independently by each researcher.

### 2.4. Quality Assessment

The quality assessment of the included studies was conducted by applying the Newcastle–Ottawa scale. According to the scale, the studies are evaluated on the basis of the following three categories: (1) selection of groups to be studied, (2) comparability, and (3) outcome (cohort studies), or selection of groups (for the purpose of avoiding selection bias), comparability and risk factor exposure (case–control studies). When a study fulfills a criterion, it is awarded a star. A total of four stars may be awarded to the selection category, two to the comparability category and three to the outcome/exposure category. The studies are then rated as low quality (<6 stars), medium quality (6–7 stars), or high quality (8–9 stars). Table 1 presents the evaluation of the studies included in this systematic review [34,35,36,37,38,39,40,41,42,43,44,45,46,47,48,49,50,51,52,53,54,55,56,57,58]. 

## 3. Results

**Figure 2:**
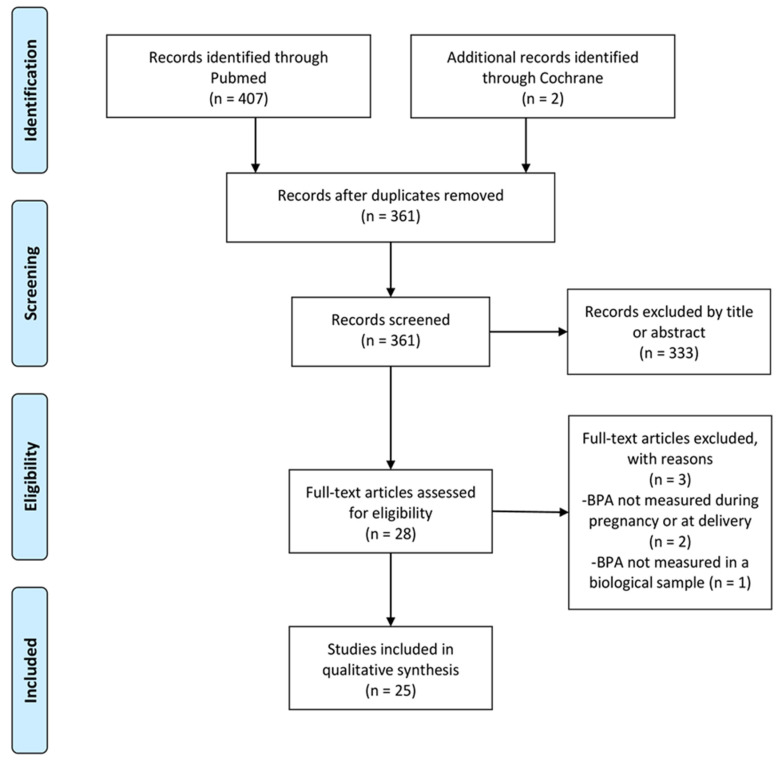
Prisma flow diagram of the selection process.

Overall, we identified 409 results (PubMed: 407 results, Cochrane: 2 results). After removal of duplicates, i.e., articles that appeared more than once during the literature search because of the utilization of more than one search term, 361 articles remained, which were screened: of these, 333 were excluded because their titles or abstracts were unrelated to the subject. Three more were excluded due to their not meeting the eligibility criteria (Table 2) [60,61,62], with, finally, 25 articles remaining for the qualitative analysis (Table 3) [34,35,36,37,38,39,40,41,42,43,44,45,46,47,48,49,50,51,52,53,54,55,56,57,58]. Figure 2 presents the process that followed (PRISMA flow diagram).

### 3.1. Fetal Growth Studies Examining First- and Second-Trimester Exposure

Early pregnancy can be a critical period, given that the fetal detoxification system is not yet fully developed. Five studies measured BPA in biological samples collected during the first or second trimester, namely this crucial stage. One of the latter groups of researchers measured this endocrine-disrupting chemical (EDC) in the early second trimester in maternal plasma and amniotic fluid of 52 non-smoking women carrying fetuses with a normal karyotype. They found no relationship between BPA levels in plasma (median: 8.69 ng/mL) or either in amniotic fluid (median: 1.03 ng/mL); they also showed that while BPA levels in plasma were not correlated with birth weight, the BPA permeability factor (a ratio of fetal-to-maternal BPA concentration) was negatively associated with this growth indicator (R  =  −0.54, *p*  <  0.001) [34]. Meanwhile, however, another team, using as a biological sample second-trimester amniotic fluid of mothers with singleton-term pregnancies, demonstrated that concentrations between 0.40 and 2.0 ng/mL result in markedly lower birth weight (−241.8 g, *p* = 0.049). The authors concluded that BPA has a non-monotonic effect and that low-level exposure can reduce birth weight [35]. 

A case–referent study of 69 cases and 69 referent pools derived from 550 case–referent pairs of full-term singleton pregnancies reported that a comparison between neonates below the 10th percentile of estimated weight for gestational age and neonates who were appropriate for gestational age exhibited no difference in BPA concentrations (mean concentration: 0.5 ng/mL) in maternal serum at 15–16 weeks of pregnancy; hence, no relationship with fetal growth restriction was found [36]. Another group of researchers, in two different studies, measured BPA using a single maternal urine sample in pregnancies with a male fetus at the end of the second and at the beginning of the third trimester. No significant association with birth weight was observed in either of these studies despite the use in the first one of multiple US scans [37,38]. Even though two out of five studies found an inverse association, the fact that they used amniotic fluid in the early second trimester, which reflects the composition of fetal plasma [63,64], raises doubts as to whether associations between BPA and lower birth weight exist. This is amplified by the fact that the other two studies investigated this relationship only in pregnancies with male fetuses. Furthermore, the latter study applied a different approach for examining exposure, as pooled maternal sera were used, with each pool containing eight to nine individual serum samples of 250 µL each; again, this study failed to show any heterogeneity of exposure within an individual pool [36].

### 3.2. Fetal Growth Studies Examining Third-Trimester Exposure and Delivery

Twelve studies measured BPA in the third trimester or at delivery. Researchers who conducted two studies as part of a prospective birth cohort study in Korea during the third trimester found a positive association of BPA in maternal urine with birth weight, particularly in male fetuses, a positive association with ponderal index values in particular in female fetuses, and decreased third-trimester femoral length in both sexes: in other words, they demonstrated an increase in BPA concentration by 1 log-transformed unit of BPA/Cr led to reduced femur length by 0.03 cm in all fetuses and by 0.06 in fetuses with specific maternal glutathione transferases (GSTs) polymorphisms [39,40]. A large number of studies have also measured BPA at delivery. While no significant correlation between BPA in cord blood (mean concentration: 48.3 ± 2.2 ng/mL) and birth weight was reported in one cross-sectional study of 187 healthy newborns (9 SGA, 5 LGA) [41], in another study, a negative association of BPA in the highest quartile (>7.04 ng/mL) in maternal and umbilical cord blood (mean concentration: 0.5 ng/mL) with birth weight and an increased risk for LBW and SGA, especially in male neonates, were observed (OR was 2.42 and 2.01, respectively). The results showed a non-monotonic dose–response curve [42]. Other authors measured BPA in maternal and cord blood from 90 mothers from antenatal clinics located in a region with high environmental pollution and found a positive association of cord blood BPA with birth weight [43], whereas another reported no association of BPA measured in cord blood and placental sample [44]. 

When BPA was measured in maternal urine of 620 women from China (mean concentration: 1.24 ng/mL) investigating its relationship with birth weight and gestational diabetes mellitus, reduced risk for this metabolic disease and decreased birth weight were found, a result that was borderline and not statistically significant (each unit increase in natural log-transformed BPA reduced birth weight by 25.70 g (95% CI = −54.48, 3.07) and ponder index by 0.02 (95% CI = −0.03, 0.00)) [45]. A study using placental samples also strongly pointed to an inverse association between BPA and birth weight and higher concentrations of this substance in LBW and SGA neonates [46]. A case–control study reported an increased risk of LBW neonates as well as elevated concentrations of BPA in urine samples of mothers who delivered LBW babies by comparison with the control group, this noted mainly in females [47]. Yet other investigators have, by contrast, observed a positive association, or else no association, between birth size and this EDC in maternal blood or urine [48,49,50]. 

### 3.3. Fetal Growth Studies with Multiple Samples and US Scan Assessments throughout Pregnancy 

Eight studies used multiple sample collections and/or US scans during different periods of gestation. According to one of them (first trimester, cord blood, and delivery), first-trimester BPA has an inverse association with birth weight, especially in female neonates (−55 g for all and −183 g for females, for a 2-fold increase in BPA) [51]. A group of researchers (using three urine spots at 11 and 26 weeks and at delivery) also measured concurrent exposure to EDCs and concluded that BPA over the 75th percentile in the third trimester, while not affecting birth weight, leads to decreased head circumference [52]. A study conducted in a sub-fertile population and which included measurement of BPA in maternal and paternal preconception urine and maternal prenatal urine revealed an inverse association of maternal preconception BPA with birth weight and head circumference [53]. Another group of investigators collected maternal urine three times through pregnancy (at 16–20 weeks, 20–24 weeks, and 24–28 weeks) and found no association of BPA with birth weight or with SGA or LGA neonates [54]. Nor was any association with birth weight observed when BPA was measured in maternal urine and blood at 16 and 26 weeks [55]. 

Another approach adopted by certain researchers was to combine multiple urine sampling with US regularly throughout pregnancy. One team using this method examined four urine samples collected at 10, 18, 26, and 35 gestational weeks, as well as carrying out US: they found no association between BPA and estimated fetal weight (EFW) or birth weight [56]. On the other hand, another study using two urine samples from the first and the third trimester observed a negative association of BPA with femoral length and EFW at 12–20 weeks in males, while they identified a positive association with EFW and abdominal circumference at 12 weeks in girls [57]. Finally, one other study examining three maternal urine samples taken during early, mid-, and late pregnancy showed statistically significantly lower growth rates for fetal weight and head circumference with a non-monotonic pattern (−1.66 SD in birth weight (−683 g), for 2.51 μg/g < BPACB < 4.22 μg/g, −3.9 cm in head circumference for BPACB > 4.22 μg/g) [58]. 

## 4. Discussion

The findings of our study suggest that increased BPA levels may be associated with impaired fetal growth rate, especially when exposure occurs in the first half of pregnancy. However, given that the literature also includes results that contradict these findings, caution is needed. The aforementioned association concerns total exposure to BPA from combined dietary, occupational, and environmental exposure.

Overall, three studies reported a positive correlation of BPA with a growth indicator, two studies showed contradictory effects, whereas 11 failed to find a statistically significant correlation. However, the fact that 14 of the 25 studies reported impairment of normal fetal growth velocity, with a total of nine of them (11 if the two studies with contradictory effects are incorporated) clearly showing a statistically significant negative association with at least one fetal growth indicator, strongly indicates that BPA can potentially constitute a contributing factor to fetal growth restriction. On the other hand, most large-scale studies that were conducted [36,37,38,39,40,45,48,49] did not identify any association. However, this could be attributed to their methodology, since measurement of this endocrine disruptor only once through pregnancy does not represent the true exposure of either the mother or the fetus during pregnancy due to BPA’s short half-life (<6 h, complete urinary elimination of orally administered BPA <24 h) [65]; these studies are therefore vulnerable to biases. Furthermore, none of the latter studies used samples that directly reflect BPA concentration in the fetus, such as in amniotic fluid. In addition, the two large-scale studies [37,38] which were conducted in the first two trimesters examined only male fetuses, while the third [36] applied a completely different methodology, given that the researchers did not quantify BPA in individual samples but instead used pooled maternal sera: it was hence not possible to estimate exposure at an individual level within the pool.

Assuming that BPA has the potential to contribute to fetal growth restriction, it would be of great importance to determine whether there is a period of increased susceptibility for the fetus during which exposure to BPA should definitely be avoided. Six of the studies that included a measurement of BPA before the third trimester [34,35,51,53,57,58] and four that did so during the third trimester or at delivery reported a negative association with an important indicator of impaired growth rate, such as birth weight, estimated fetal weight, or increased risk for LBW or SGA neonates. 

Actions that reduce estimation bias due to the short half-life of BPA [58] were undertaken in six of the 13 studies conducted before the third trimester; these steps classify them as studies of high quality. The studies in question showed an inverse correlation with fetal growth, revealing that the first half of pregnancy is highly likely to be the period of increased vulnerability for fetuses. Specifically, two of these studies collected amniotic fluid as the biological sample, two used multiple samples through pregnancy, and two used multiple samples combined with US scans.

Moreover, four of the five studies that identified an increase in a growth parameter (3 + 2 with contradictory results) collected a sample in the third trimester or at delivery [39,40,43,48]. Furthermore, in studies that investigated gender-specific effects, no particular pattern was identified. 

Taking into account only studies that took a sample directly reflecting the concentration of BPA in the fetus, i.e., amniotic fluid, cord blood, or placenta, we found that four studies showed an inverse association with birth weight, one a positive relation, and three no significant association. When we concentrated on early pregnancy, both studies that collected amniotic fluid demonstrated an inverse association with birth weight, though mediated in a non-monotonic order, strongly indicating that this is likely to be the most vulnerable period for the fetus.

The above results closely correspond with outcomes of other studies that have investigated parameters capable of affecting the growth rates of fetuses. Specifically, bisphenol A has been associated with increased oxidative stress and inflammation biomarkers [66,67], conditions that can induce adverse birth outcomes, such as fetal growth restriction [68,69,70]. Additionally, the fact that diethylstilbestrol, another xenoestrogen, can induce growth restriction raises concerns as to whether BPA can have the same outcome [71]. 

Pathophysiologically, several studies have focused on the pathways that could disrupt growth. Specifically, suspicion has been raised of impaired angiogenesis and vasculogenesis of the placenta. An association between bisphenol A in maternal urine and a rise in the sFlt-1 and sFlt-1 to PlGF ratio in maternal plasma has been observed [72]; also noted has been a reduction of vascular endothelial growth factor (VEGF), along with an impairment of trophoblast cell proliferation in first-trimester trophoblast cells exposed to BPA [8]. These changes can induce impaired angiogenesis and vasculogenesis of the placenta, leading to inadequate nutrient supply and fetal growth restriction [68,73]. Mice exposed to endocrine disruptors in early pregnancy have also presented FGR, probably because of damage to normal remodeling of spiral arteries [74]. In addition, changes in placental micro-RNA expression, DNA methylation, and genomic imprinting have been reported [7,8]; these alterations can potentially impair fetal growth by affecting endocrine functions and oxidative stress, as well as via other pathways [9,11]. Thus, a considerable number of studies show that BPA may be capable of inducing pathophysiologic alterations, especially in early pregnancy, which can impact normal intrauterine development.

The conflicting results between studies may be attributed to their variability, given that different biological samples are collected from dissimilar populations during different periods of gestation, and, moreover, usually during only a single time frame throughout pregnancy, and this, crucially, despite BPA’s short half-life. Different techniques of quantification of BPA were also used (LC-MS/MS, GC-MS, and ELISA) with different limits of detection, which, combined with the unequal exposure of the participants in each study, resulted in significant variations in BPA levels. Furthermore, studies have shown that such methods as HPLC or ELISA possess lower specificity for accurate quantification of BPA in biological samples, including umbilical cord blood [28]. Moreover, BPA concentrations in maternal plasma may not be truly representative of the exposure of the fetus to this endocrine disruptor, as studies examining amniotic fluid have shown that exposure depends on placental BPA permeability. In addition, current studies have consistently demonstrated that BPA has a non-monotonic impact on birth weight; hence, measurement only of BPA relates solely to concentrations, while non-use of stratification can potentially conceal the compound’s true effects. Finally, because BPA presents high intraindividual variability and has a short half-life, this can lead to measurement errors. Multiple measurements in future studies can help overcome these problems and thus more accurately reveal the associations. 

### Strengths and Limitations

The present systematic review has several strengths. To our knowledge, this is the first systematic review focusing on BPA effects on fetal growth based on trimester-specific exposure, a fact that can give prominence to the period of greater susceptibility of the fetus. Moreover, a large number of studies have been included, representing a large sample size. Another important feature is that our search was not constricted to birth weight as an indicator of intrauterine growth, but also used multiple growth indicators. 

However, our review also has several limitations. Firstly, there was great heterogeneity between the included studies as concerns the studied populations, the biological samples collected, the design of the studies, and the method used for quantifying BPA, which meant that no meta-analysis was possible for this review. Second, BPA has a short half-life and, as most studies made only one measurement during pregnancy, they may contain estimation biases.

## 5. Conclusions

There is growing evidence that BPA can reduce fetal growth, especially in early pregnancy. It is therefore recommended that exposure to this endocrine disruptor should be as limited as possible during this time period. The actual cut-off value of BPA exposure that could predict abnormalities of fetal growth remains as yet unknown. Moreover, given that combined exposure to dietary and non-dietary sources cannot be considered safe, it certainly seems reasonable to question whether usual environmental exposure during early pregnancy should be regarded as potentially hazardous. In view of the conflicting results of the included studies, more studies are needed in the field to fully elucidate the role of BPA in fetal growth. Although recent studies have contributed significantly to our knowledge about BPA, more are required focusing on its sex-specific effects and, importantly, examining during which gestational period the fetus is most susceptible. Future studies need to incorporate multiple collections of biological samples as well as US and Doppler scans to be carried out throughout pregnancy, and especially during the first half. Such an endeavor will not only reduce the risk of bias, thus helping to shed further light on the intrauterine development of exposed fetuses, but will also pinpoint the critical window of vulnerability. 

## Figures and Tables

**Table 1:** Quality assessment of the included studies based on the Newcastle–Ottawa scale.

Study	Selection	Comparability	Outcome/Exposure	Total Score
Krętowska et al., 2019 [34]	***	**	***	8
Pinney et al., 2017 [35]	***	**	***	8
Burstyn et al., 2013 [36]	****	**	***	9
Phillipat et al., 2014 [37]	***	**	***	8
Phillipat et al., 2019 [38]	***	**	***	8
Lee et al., 2014 [39]	***	**	***	8
Lee et al., 2018 [40]	***	**	***	8
Dalkan et al., 2019 [41]	***	*	***	7
Chou et al., 2011 [42]	***	**	***	8
Gounden et al., 2019 [43]	***	*	***	7
Xu et al., 2015 [44]	**	*	***	6
Wang et al., 2017 [45]	***	**	***	8
Troisi et al., 2014 [46]	***	**	***	8
Huo et al., 2015 [47]	****	**	***	9
Ding et al., 2017 [48]	***	**	***	8
Tang et al., 2013 [49]	***	**	***	8
Padmanabhan et al., 2008 [50]	***	**	***	8
Veiga-Lopez et al., 2015 [51]	***	*	**	7
Huang et al., 2017 [52]	***	**	***	8
Mustieles et al., 2018 [53]	**	**	***	7
Aker et al., 2019 [54]	***	**	***	8
Woods et al., 2017 [55]	***	**	***	8
Ferguson et al., 2016 [56]	***	**	***	8
Casas et al., 2016 [57]	***	**	***	8
Snijder et al., 2013 [58]	***	**	***	8

Award of the stars can be found on http://www.ohri.ca/programs/clinical_epidemiology/nosgen.pdf (accessed on 13 July 2021) [59]. In the comparability category the particular confounding factors were gestational age (most important factor) and at least one of the following: maternal age, smoking, educational level, pregestational weight, height, race and parity.

**Table 2:** Studies excluded from the review.

Author	Year	Reason for Exclusion
Bell et al., 2018 [60]	2018	BPA not measured during pregnancy or at delivery
Smarr et al., 2015 [61]	2015	BPA not measured during pregnancy or at delivery
Miao et al., 2011 [62]	2011	BPA not measured in a biological sample

**Table 3:** Studies on the association of BPA with fetal growth.

Study	Participants—Country—Type of Study	Biological Sample—Time—Method of Detection—Detection Limit/Limit of Quantification	Median Concentration-50th Percentile/Mean Concentration/	BW/EFW	BL/WFL	SGA/LBW	AC/FL/HC/BPD	PI	PW/PBWR
Krętowska et al., 2019 [34]	52—Poland cohort study	Maternal blood, amniotic fluid—between 15 and 18 weeks—GC-MS	8.69 ng/mL (plasma), 1.03 ng/mL (amniotic fluid)/-	↓ BPA permeability factor—BW (R = −0.54, *p* < 0.001)					
Pinney et al., 2017 [35]	130—USA cohort study	Amniotic fluid—between 16 and 22 weeks (mean 17.2)—LC-ECAPCI-MS/MS—0.08 ng/mL/0.25 ng/mL	0.36 ng/mL/-	↓ 241.8 g (group with 0.41–2.0 ng/mL BPA compared to group with ≤0.25 ng/mL					
Burstyn et al., 2013 [36]	1100 –Canada case–control study	Maternal serum—15–16 weeks—APCI-MS/MS—0.1 ng/mL	-/0.5 ng/mL (mean difference: 0 ng/mL)	-					
Phillipat et al., 2014 [37]	520—France cohort study	Maternal urine -between 22 and 29 weeks—0.4 ng/mL	2.4 ng/mL/-	-/-	-		-/-/-/-		
Phillipat et al., 2019 [38]	473—France cohort study	Maternal urine—between 23 and 29 weeks—online solid phase extraction—HPLC-electrospray ionization-tandem mass spectrometry—0.4 ng/mL	2.34 ng/mL/-	-					-/-
Lee et al., 2014 [39]	757—Korea cohort study	Maternal urine—28–42 weeks—HPLC-isotope dilution tandem mass spectrometry—0.12–0.28 ng/mL	1.08 ng/mL (1.63 μg/g creatinine)/1.29 ng/mL (1.87 μg/g creatinine)	↑ 66.9 g in ♂ (second tertile compared to first)	-			↑ 0.12 g/cm^3^ × 100 in ♀ (r = 0.11)	
Lee et al., 2018 [40]	788—Korea cohort study	Maternal urine—third trimester Neonatal urine—HPLC-isotope dilution tandem mass spectrometry −0.12–0.28 ng/mL	-/1.26 μg/g Cr	↑ in BPA by 1 log-transformed unit of BPA/Cr: ↑ z-score 0.05 and 0.06 in ♂/-	↑ in BPA by 1 log-transformed unit of BPA/Cr: -/↑ 0.05 0.07 in ♀		↑ in BPA by 1 log-transformed unit of BPA/Cr: ↓ FL 0.03 cm and 0.06 cm (GSTs)		
Dalkan et al., 2019 [41]	150—Cyprus cohort study	Cord blood—delivery—sandwich enzyme-linked immunosorbent assays (ELISA)—	-/48.3 ± 2.22 ng/mL	-/	-/		- (HC)		
Chou et al., 2011 [42]	97—Taiwan cohort study	Maternal blood, umbilical cord blood—delivery—HPLC/UV detector—0.13 ng/mL	-/2.5 ng/mL (maternal blood) and 0.5 ng/mL (umbilical cord blood)			↑ (OR = 2.01)/ ↑ (OR = 2.42)			
Gounden et al., 2019 [43]	90—South Africa cohort study	Maternal blood, umbilical cord blood—third trimester –ultra HPLC–MS/MS—0.12 ng/mL	Maternal blood: BPA (0.95 ng/mL), BPA-glucuronide (4.71 ng/mL), cord blood: BPA (0.92 ng/mL), BPA-glucuronide (4.21 ng/mL)/-	↑ (cord blood BPA)/					
Xu et al., 2015 [44]	200—China cohort study	Cord blood, placenta—delivery—GC/MS	6.369 ng/mL (exposed group) 2.824 ng/mL (reference group)/-	-/	-/				
Wang et al., 2017 [45]	620—China cohort study	Maternal urine—delivery -HPLC-MS/MS—0.1 ng/mL	-/1.32 ng/mL	-/				-	
Troisi et al., 2014 [46]	200—USA case–control study	Placenta—delivery—isotope dilution GC-MS	-/103.4 ± 61.8 ng/g	-/↓ CBWC for ↑ BPA (*p* = 0.0112, r = −0.179)		↑ BPA (157.9 ng/g)/↑ BPA (125.4 ng/g) in cases			
Huo et al., 2015 [47]	452—China case–control study	Maternal urine—delivery—UPLC–MS/MS—0.2 ng/mL	4.70 ng/mL (cases) 2.25 ng/mL (controls) *p* < 0.05/-			-/↑ Risk, OR = 3.13 for the medium tertile, OR = 2.49 for the highest tertile			
Ding et al., 2017 [48]	496—China cohort study	Maternal urine—delivery—HPLC-MS/MS—0.1 ng/mL	0.48 ng/mL, 1.07 μg/g creatinine/-	-	10-fold ↑ in BPA ↑ 0.63 cm in ♂/-		- (HC)	-	
Tang et al., 2013 [49]	567—China cohort study	Maternal urine—delivery—UPLC–MS/MS—0.36 ng/mL	-/0.91 ng/mL	-/	-/				
Padmanabhan et al., 2008 [50]	40—USA cohort study	Maternal blood—delivery—HPLC-MS/MS—0.5 ng/mL	-/5.9 ng/mL	-/					
Veiga-Lopez et al., 2015 [51]	80—USA cohort study	Maternal blood— 8—14 weeks and delivery Umbilical cord blood—delivery—HPLC-MS/MS—Phase 1: 0.05 ng/mL, Phase 2: 0.02 ng/mL	-/Phase 1: Maternal blood (8–14 weeks: 1.0 ng/mL, delivery 1.7 ng/mL, umbilical cord blood 0.5 ng/mL Phase 2: Maternal blood (8–14 weeks: 4.8 ng/mL, delivery 11.9 ng/mL, umbilical cord blood 3.1 ng/mL	↓ (−55 g and −183 g in ♀ for 2-fold increase in BPA (8–14 weeks)/					
Huang et al., 2017 [52]	162—Taiwan cohort study	Maternal urine—11, 26 weeks and delivery- time-of-flight mass spectrometer with an electrospray interface and UPLC—0.16 ng/mL	/11 weeks: 0.17 μg/g creatinine, 26 weeks: 0.37 μg/g creatinine, delivery: 0.34 μg/g creatinine	-/	-/		↓ (HC) −0.52 cm (3rd trimester)		
Mustieles et al., 2018 [53]	346—USA cohort study	Maternal and paternal urine (preconception, 6, 21 and 35 weeks)—HPLC-MS/MS—0.4 ng/mL	/1.6 ng/mL (paternal), 1.5 ng/mL (maternal preconception) and 1.2 ng/mL (maternal prenatal)	each ln unit ↑ in BPA: ↓ 119 g (maternal preconception)/			↑ in BPA: ↓ HC 0.72 cm (maternal preconception)		
Aker et al., 2019 [54]	922—Puerto Rico cohort study	Maternal urine—16–20, 20–24 and 24–28 weeks—HPLC-MS/MS—0.2 ng/mL	/2.02 ng/mL (all), 2.16 ng/mL (16–20 weeks), 2.07 ng/mL (20–24 weeks), 1.78 ng/mL (24–28 weeks)	-/					
Woods et al., 2017 [55]	272—USA cohort study	Maternal urine, maternal blood—16 and 26 weeks/LC -MS or GC-MS—0.4 μg/g	/2.1 μg/g	-/					
Ferguson et al., 2016 [56]	482—USA cohort study	Maternal urine—at median 10, 18, 26, and 35 weeks, LC-MS/MS	1.28 ng/mL (10 weeks), 1.33 ng/mL (18 and 26 weeks), 1.32 ng/mL (35 weeks)/	-/-			-/-/-/		
Casas et al., 2016 [57]	470—Spain cohort study	Maternal urine—12 and 32 weeks—LC-MS-0.1 ng/mL, 0.1 μg/g (creatinine-adjusted)	-/2.3 ng/mL, 2.6 μg/g	-/↓ (–5.74% SD) in ♂ (12 to 20 weeks), ↑ in ♀ (12 weeks)—not creatinine-adjusted			↑ (6.41% SD) in ♀ (12 weeks)/↓ in ♂ (12 to 20 weeks)/-/-		
Snijder et al., 2013 [58]	219—Netherlands cohort study	Maternal urine—13, 21 and 30 weeks—tandem mass spectrometry—0.26 ng/mL, 0.05 ng/mL	-/1.7 µg/g, 3.2 µg/g	-/↓ (–683 g)			-/-/↓ (–3.9 cm)/-		

Abbreviations: BW, birth weight; EFW, estimated fetal weight; BL, birth length; WFL, z-score for weight-for-length at birth; SGA, small for gestational age; LBW, low birth weight; AC, abdominal circumference; FL, femur length; HC, head circumference; BPD, biparietal diameter; PI, ponderal index; PW, placental weight; PBWR, placental-to-birth weight ratio; CBWC, calculated birth weight centile; GC-MS, gas chromatography-mass spectrometry; LC-ECAPCI-MS/MS, liquid chromatography-electron capture atmospheric pressure chemical ionization-tandem mass spectrometry; APCI-MS/MS, atmospheric pressure chemical ionization and tandem mass spectrometry; HPLC, high-performance liquid chromatography; HPLC-MS/MS, high-performance liquid chromatography–tandem mass spectrometry; UPLC–MS/MS, ultra-performance liquid chromatography–tandem mass spectrometry; glutathione transferases, GST; ♂, males; ♀, females; ↑: Outcome was increased in the presence of BPA; ↓: outcome was decreased in the presence of BPA; —: outcome was not influenced by the presence of BPA; blank boxes indicate that the outcome was not investigated.
